# Scientific Items

**Published:** 1883-09-20

**Authors:** 


### SCIENTIFIC ITEMS.

**Copperhead Venom.**—Dr. I. Ott, (Virginia Medical Monthly, February, 1883), comes to the following conclusions:

1. The venom of the copperhead is weaker in toxic activity than that of the rattlesnake.

2. The heart, with both kinds of venom, becomes greatly prostrated, and in rapid deaths is their main cause.

3. The venom of either snake does not affect the sensory nerves.

4. The sensory centers are affected by both venoms.

5. The muscular excitability continues to be little affected at the time of death by the poison of the copperhead.

6. The two venoms greatly resemble each other in physiological activity.

7. The cardiac force, rythm, and frequency are lowered by both venoms.

8. The arterial tension is greatly lowered by both venoms.

9. The blood, after copperhead-poisoning, shows no microscopic changes of its globules, and no difference in its spectrum.—*Med. and Surg. Rep.*

**The Inventor of the Telephone.**—The assertion that Bell is not the inventor of the telephone, and the fact that proof positive exists to fully sustain the assertion, is a matter of no ordinary interest to every intelligent reader. Prof. S. B. Thompson, of England, has very thoroughly investigated the inventions and researches of Johann Phillipp Reis, a German; and he finds that this man discovered the electric transmission of speech in 1860–'61, and that he used devices and instruments corresponding with those now used in what is known as the Bell system of telephony.

The full establishment of the facts as presented crushes the Bell telephone monopoly as with the weight of a mountain. The telephone becomes the property of the world, open and free to all. This is a consummation devoutly to be wished for. It is true, as observed by Dr. Channing, "there is a menace in connection with its present control, which justly awakens public concern. Rapacious hands have clutched the throat of the telephone to extort oppressive tribute for every word which it utters. In the light of historic facts, the decision of the courts of the United States, that Bell is the discoverer of a new and useful art (the electric transmission of speech), to which he has exclusive title *must be reversed* as speedily as possible, that our courts may retain the respect of the people of the United States."—*Pop. Sci. News.*

**Showers of Iron.**—In the News for July, we referred to the occurrence of iron in meteoric dust. Inquiries have since been addressed to us concerning the phenomenon; and a few additional words on the subject may be of interest to our readers in general.

On the night of the 29th of March, 1880, there was a fall of meteoric dust, accompanied with rain, at Catania, in Sicily. This dust, besides having the red color, mineral. and organic particles, and minute infusoria, frequently observed before on similar occasions, was especially interesting, because it contained a considerable quantity of iron, either in a pure metallic state, or in metallic particles surrounded by an oxidized crust. The fragments were of sizes varying from one to ten hundredths of a millimeter in diameter. Some were of an irregular, others, of a perfectly spherical shape, as if they had been suddenly fused. All were immediately attracted by the magnet. This fact (discovered for the first time in dust gathered on board of a ship in the Indian Ocean on the night of the 24th of January, 1849, and afterwards confirmed by Professor Nordenskjold on the "Vega" in the Arctic and other seas), as a scientific writer remarked at the time, is "of immense importance to physical and geological science, as proving that iron, which is not known in a pure metallic state on the surface of the earth, is to be regarded as of extra-terrestrial or cosmic origin, establishing a link between the earth and the chaotic material dispersed over the universe, and as being also in strict relation with the phenomena of aerolites and meteors."—*Pop. Sci. News.*

**Household Ventilation.**—Dr. Russell, in the Glasgow Health Lectures, says of the ventilation of the house-rooms :

"Minimize as we may the progressive contamination of an enclosed inhabited space, the contamination is still progressive, and, without renewal of the air, in a few hours you will reach the boundary beyond which lies impaired health. Open your windows, pull up your window blinds, turn up your mattresses and bedclothes, and every morning let the products of the night be swept out by the incoming current of fresh air. Then, all through the day, remember to have a small chink open at the top of your windows ; or, better still, raise the lower sash, close the opening beneath with a piece of wood fitting closely, and so the air will enter at the junction of the sashes, and pass upwards without draught. The secret of ventilation without draught is a little and constantly. Once permit the air to become close and stuffy, and, the moment you endeavor to remedy this result of carelessness, a cold draught will rush in, and the fear of injury will prompt you to stop it. The mere fact of living in a close atmosphere begets a shivery, susceptible condition of the body, which is intolerant of the slightest sensation of chill. If you accustom yourself and your children to fresh air, the vital heat is sustained, and even a draught becomes exhilarating.—*Ibid.*

**Variety of Colors.**—"About fifteen thousand varieties of color are employed by the workers of mosaic in Rome, and there are fifty shades of each of these varieties, from the deepest to the palest ; thus affording seven hundred and fifty thousand tints, which the artist can distinguish with the greatest facility."—*Ex.*

